# *QuickStats:* Death Rates* from Septicemia^†^ Among Persons Aged ≥65 Years, by Age Group — National Vital Statistics System, United States, 2000–2018

**DOI:** 10.15585/mmwr.mm6946a7

**Published:** 2020-11-20

**Figure:**
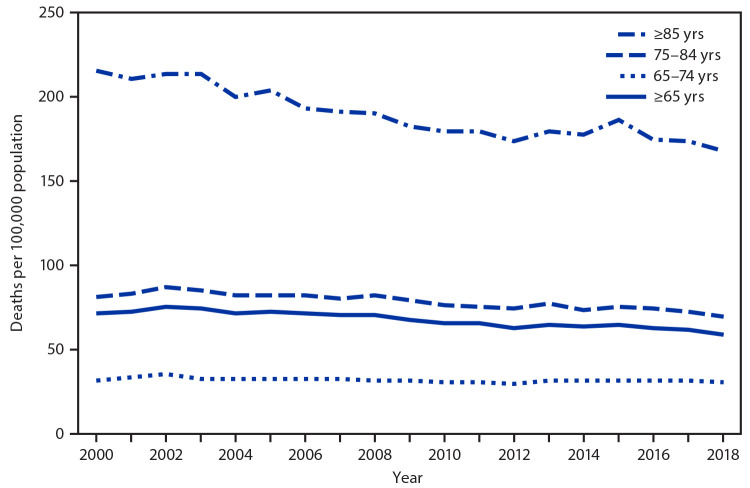
During 2000–2018, the death rate from septicemia among persons aged ≥65 years generally decreased from 70.8 to 58.7 deaths per 100,000 population. The death rate was lower in 2018 than in 2000 among persons aged 75–84 years (80.4 compared with 69.4) and among persons aged ≥85 years (215.7 compared with 167.4). The death rate for persons aged 65–74 was similar in 2000 (31.0) and 2018 (30.0). In each year during 2000–2018, the death rate was highest among persons aged ≥85 years and lowest among persons aged 65–74 years.

**Source:** National Vital Statistics System mortality data. https://www.cdc.gov/nchs/nvss/deaths.htm.

